# Association testing by haplotype-sharing methods applicable to whole-genome analysis

**DOI:** 10.1186/1753-6561-1-s1-s129

**Published:** 2007-12-18

**Authors:** Ilja M Nolte, André R de Vries, Geert T Spijker, Ritsert C Jansen, Dumitru Brinza, Alexander Zelikovsky, Gerard J te Meerman

**Affiliations:** 1 Department of Epidemiology, University Medical Center Groningen, University of Groningen, Hanzeplein 1, 9713 GZ Groningen, the Netherlands; 2 Department of Genetics, University Medical Center Groningen, University of Groningen, Hanzeplein 1, 9713 GZ Groningen, the Netherlands; 3 Deparment of Dermatology, University Medical Center Groningen, University of Groningen, Hanzeplein 1, 9713 GZ Groningen, the Netherlands; 4 Groningen Bioinformatics Center, Groningen Biomolecular Sciences and Biotechnology Institute, University of Groningen, Kerklaan 30, 9751 NN Haren, the Netherlands; 5 Department of Computer Science, Georgia State University, 34 Peachtree Street, Atlanta, Georgia 30303-3086, USA

## Abstract

We propose two new haplotype-sharing methods for identifying disease loci: the haplotype sharing statistic (HSS), which compares length of shared haplotypes between cases and controls, and the CROSS test, which tests whether a case and a control haplotype show less sharing than two random haplotypes. The significance of the HSS is determined using a variance estimate from the theory of U-statistics, whereas the significance of the CROSS test is estimated from a sequential randomization procedure. Both methods are fast and hence practical, even for whole-genome screens with high marker densities. We analyzed data sets of Problems 2 and 3 of Genetic Analysis Workshop 15 and compared HSS and CROSS to conventional association methods. Problem 2 provided a data set of 2300 single-nucleotide polymorphisms (SNPs) in a 10-Mb region of chromosome 18q, which had shown linkage evidence for rheumatoid arthritis. The CROSS test detected a significant association at approximately position 4407 kb. This was supported by single-marker association and HSS. The CROSS test outperformed them both with respect to significance level and signal-to-noise ratio. A 20-kb candidate region could be identified. Problem 3 provided a simulated 10 k SNP data set covering the whole genome. Three known candidate regions for rheumatoid arthritis were detected. Again, the CROSS test gave the most significant results. Furthermore, both the HSS and the CROSS showed better fine-mapping accuracy than straightforward haplotype association. In conclusion, haplotype sharing methods, particularly the CROSS test, show great promise for identifying disease gene loci.

## Background

With the current advances in genotyping technology, genome-wide association studies for detecting genes involved in complex diseases have recently become feasible. However, the computational and statistical methodology for analyzing such studies needs optimization and standardization. Various methods and strategies have been investigated [1-3]. The typical association study of candidate regions uses single locus tests, such as the chi-square test or the transmission disequilibrium test [4]. The advantage of single-locus tests is that haplotype inference can be avoided. However, it is expected that there is more information contained in haplotypes as a result of the underlying evolutionary processes [5,6]. Therefore, association analysis based on haplotypes is believed to be more powerful, in particular if the common disease-common variant theory applies. According to this, the genetic variants related to a complex disease are old mutations and are common in the population (minor allele frequencies > 5%). Subsequent mutations and recombinations in the ancestral haplotype at which the disease mutation occurred shortened the haplotypes that descended from this ancestor. However, in the current generation the haplotypes will still share a fragment around the disease locus. The size of the reduced ancestral haplotype fragments varies as a result of the uneven nature of such recombination and mutation processes. We hypothesize that there is a difference in haplotype patterns between cases and controls at regions associated with the disease and we present two new methods based on this hypothesis: the haplotype-sharing statistic (HSS), which is an improvement of a statistic described by Van der Meulen et al. [7,8], and the CROSS test, which was published in an earlier form [9]. The test statistics of these two haplotype-sharing methods will be described and their performance will be compared to standard association methods.

## Methods

### Materials

Data from both Problems 2 and 3 of Genetic Analysis Workshop 15 (GAW15) were analyzed. From Problem 2, we used a data set of the North American Rheumatoid Arthritis Consortium (NARAC) of 2300 single-nucleotide polymorphisms (SNPs) covering 10 Mb of chromosome 18q, a region that had shown linkage evidence for rheumatoid arthritis (RA) [10]. This data set contained 460 cases and 460 controls, the latter being recruited from a New York City population. Problem 3 provided simulated RA data on 9187 SNPs distributed over the entire genome. We used Replicates 1 through 10 of the affected sib-pair nuclear families for analysis. From each family, haplotypes transmitted to the first affected sib were used as cases and non-transmitted haplotypes as controls, regardless of the affection status of the parents. We had no prior knowledge of the answers.

### Haplotype inference

The 920 subjects of the NARAC data set were phased using the phasing program 2SNP [11]. This program reconstructed the 1840 haplotypes for all 2300 SNPs in approximately 20 minutes. The construction of the simulated data sets allowed extraction of phased haplotypes and these data sets were analyzed without phase ambiguities or missing alleles.

### Statistical analysis

Haplotype sharing between two haplotypes *X *and *Y *from the perspective of each locus *k*, denoted as *h*(*X*, *Y*; *k*), can be evaluated as the number of consecutive SNPs in the telomeric and centromeric directions carrying the same alleles including locus *k*. Given a sample of case haplotypes *X*_1_,...,*X*_*N *_and a sample of control haplotypes *Y*_1_,...,*Y*_*M*_, four measures of haplotype sharing at locus *k *are defined as follows:

case sharing: $SH^{CASE}(k)=\frac{2}{N(N-1)}\cdot \sum_{i=1}^{N-1}\sum_{j=i+1}^{N}h(X_{i},X_{j};k)$;

control sharing: $SH^{CTR}(k)=\frac{2}{M(M-1)}\cdot \sum_{i=1}^{M-1}\sum_{j=i+1}^{M}h(Y_{i},Y_{j};k)$;

cross sharing: $SH^{CROSS}(k)=\frac{1}{MN}\cdot \sum_{i=1}^{N}\sum_{j=1}^{M}h(X_{i},Y_{j};k)$;

overall sharing: $SH^{ALL}(k)=\frac{N^{2}\cdot SH^{CASE}(k)+M^{2}\cdot SH^{CTR}(k)+2NM\cdot SH^{CROSS}(k)}{{\left(N+M\right)}^{2}}$.

The first haplotype sharing method, the HSS, compares the case haplotype sharing with control haplotype sharing. In contrast to the earlier HSS [7,8], in this manner the HSS corrects for linkage disequilibrium (LD) other than that caused by the disease mutation. We hypothesize that haplotype sharing will be larger among cases than among controls at loci involved in the disease and at other loci in LD with them, because i) haplotypes containing a risk allele are more likely to be similar to each other and dissimilar to haplotypes containing a non-risk allele; and ii) haplotypes containing the risk allele may be shared over longer stretches. The first factor is explained from the concepts of association and LD. The second factor can be explained by presumably shorter coalescence times of disease alleles and hence fewer recombination events in the sample of cases compared to the sample of wild-type alleles in controls. The HSS at locus *k *is defined as

$$t_{HSS}(k)=\frac{SH^{CASE}(k)-SH^{CTR}(k)}{\sqrt{{\left(sdSH^{CASE}(k)\right)}^{2}+{\left(sdSH^{CTR}(k)\right)}^{2}}},$$

where *sdSH*^*CASE*^(*k*) and *sdSH*^*CTR*^(*k*) are the estimates of the standard deviation of the mean haplotype sharing at locus *k *accounting for LD among cases and controls, respectively. When *N *and *M *are large, *SH*^*CASE*^(*k*) and *SH*^*CTR*^(*k*) follow a normal distribution (Central Limit Theory) and, because *SH*^*CASE*^(*k*) and *SH*^*CTR*^(*k*) are independent, significances of *t*_*HSS*_(*k*) can be derived from a *t*-distribution with *N *+ *M *- 2 degrees of freedom (i.e., *N *- 1 from the cases and *M *- 1 from the controls). The main statistical problem in evaluating mean haplotype sharing is how to calculate the variance of the mean sharing between all pairs of haplotypes. Generally, haplotypes will share alleles in groups and this means that haplotype agreement between haplotype pairs is not independent. For the HSS, we derived an unbiased estimate from the theory of U-statistics for the standard deviation of the mean haplotype sharing (see Appendix).

The second haplotype-sharing method is the CROSS test. This hypothesizes that a case and a control haplotype are different from each other in the region of a disease locus and will therefore show less haplotype sharing (cross sharing; *SH*^*CROSS*^) than two random haplotypes (*SH*^*ALL*^). This test incorporates more information on allele frequency differences between cases and controls (i.e., the single SNP association "signal") than the HSS. Unlike the HSS, an equivalent U-statistics variance of the cross sharing can not be estimated because of the correlation between *SH*^*CROSS *^and *SH*^*ALL*^. Therefore, the variance of the cross sharing is estimated from a sequential randomization procedure in which case and control status is randomly permuted over the haplotypes as long as the interim significance estimate remains interesting (i.e., *p*-value < 0.1). In order to render this test fast and hence feasible for whole-genome screens with a high density of SNPs, the significance is not determined from the randomization procedure, but the variance of *SH*^*CROSS*^(*k*) - *SH*^*ALL*^(*k*) is estimated from a maximum of 200 randomizations, which is a sufficient number to provide a reasonably accurate variance estimate. The CROSS test at locus *k *is then defined as

$$z_{CROSS}(k)=\frac{SH^{CROSS}(k)-SH^{ALL}(k)}{sd(SH^{CROSS}(k)-SH^{ALL}(k))}.$$

Note that a negative value implicates positive association. As a result of the correlation between *SH*^*CROSS *^and *SH*^*ALL*^, the tails of the *z*_*CROSS*_(*k*) distribution are not properly approximated by a normal distribution, leading to downward biased *p*-values for extreme *z*-values. Therefore, the *z*-values are transformed to a chi-square distribution with *ν *degrees of freedom:

$$P(\chi^{2}|\nu )\sim \Phi (z)\;where\;z=\frac{\chi^{2}-\nu }{\sqrt{2\nu }}\Rightarrow \chi_{CROSS}^{2}(k)\sim z_{CROSS}(k)\cdot \sqrt{2\nu }+\nu .$$

With an appropriately chosen *ν*, the distribution resembles the true *z*-score distribution, especially in the tails, so that realistic *p*-values are obtained. The best choice for *ν *typically depends on the sample size and on the individual chromosome. For the current study, we empirically derived the value for *ν *that minimized the bias in *p*-values in non-associated regions.

In order to compare the performances of the HSS and CROSS, we also performed single-SNP and haplotype-association analysis. Single-SNP association was tested by means of a chi-square test. For haplotype association, frequencies of haplotypes of five consecutive SNPs were counted and a log-likelihood ratio test was performed including only haplotypes with *n *> 10 to assess the significance of the difference between cases and controls (our own software, available on request).

We used a conservative Bonferroni correction to correct for multiple testing. Hence, in the real data, a result was considered significant if the -log(*p*-value) was larger than -log(0.05/2300) = 4.65, and in the simulation study if the -log(*p*-value) was larger than -log(0.05/9187) = 5.25 and suggestive if it was larger than -log(0.10/9187) = 4.95.

## Results

### NARAC data set (Problem 2)

The running time of all analyses for 2300 SNPs and 920 individuals was 80 minutes on a single laptop PC (Celeron 1500 MHz), which is sufficiently fast to be acceptable for whole-genome association studies.

**Figure 1:**
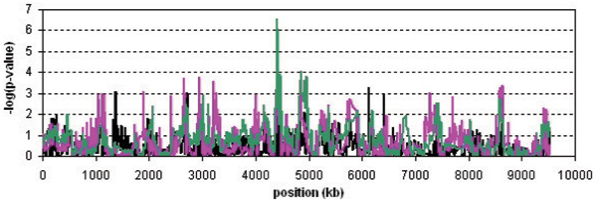
**Analysis results of the NARAC data set**. Results of the CROSS test (green line), the HSS (pink line) and single-marker association (black line) are plotted for all 2300 SNPs on chromosome 18q.

**Figure 2:**
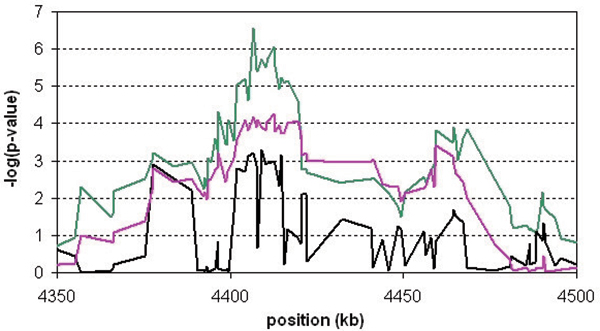
**Region showing the strongest association in the NARAC data set**. The CROSS (green line), the HSS (pink line) and single-SNP association (black line) -log(*p*-values) are plotted for the 150-kb region showing the strongest association.

The results are presented in Figures 1 and 2. The CROSS test revealed a significant association with a -log(*p*-value) of 6.6 at position 4407 kb from the first SNP. The odds ratio based on allele frequencies at this location is 1.41 (1.16–1.72, 95% CI). A second, non-significant peak (-log(*p*-value) = 4.1) was found near 4863 kb from the first SNP, with a corresponding odds ratio of 1.21 (0.99–1.51, 95% CI). Although the HSS peaks at the same two locations (-log(*p*) = 4.2 and 3.2, respectively), these results were not significant. The same holds for the single-marker chi-square test.

Figure 2 focuses on the associated region around 4407 kb. The CROSS test did not show a clear peak at a particular locus. Instead, several SNPs in the region were associated, which was to be expected because the CROSS test is based on haplotype sharing and tests at subsequent loci were highly correlated. However, the HSS and the single-marker chi-square test were not informative about the exact disease locus either. Nevertheless, the CROSS identified a small region of 20 kb (4,400–4,420 kb) as the candidate region.

### Simulated data set (Problem 3)

**Figure 3:**
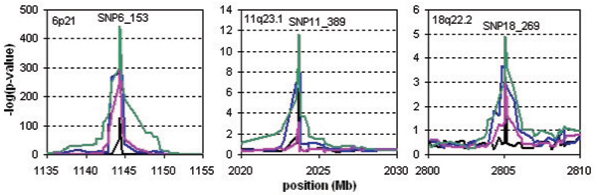
**Analysis results of the simulated data set in the regions 6p21 (a), 11q23.1 (b), and 18q22.2 (c)**. On the x-axis, the cumulative position with respect to the start of chromosome 1 is given. The black, blue, pink, and green lines represent the mean of the -log(*p*-values) of the ten replicates of single-SNP association, five-SNP haplotype association, HSS, and CROSS, respectively.

Figure 3 shows the results of the genome-wide SNP association analyses. Genome-wide significant associations were observed for all statistical methods at chromosomal regions 6p21 and 11q23.1, and we found a suggestive result at 18q22.2 for the CROSS test only. These regions are known candidate regions for rheumatoid arthritis. The CROSS test showed the most significant results: for 6p21 the mean of the -log(*p*)-values for the ten replicates was 443.6 (range 370.3–552.0); for 11q23.1 it was 22.8 (range 6.4–33.9); and for 18q22.2 it was 4.95 (range 1.8–10.6). None of the methods detected other associated regions.

Type I errors and power are shown in Table 1. Both haplotype-sharing methods had the correct type I errors. On chromosome 11 both the HSS and the CROSS tests had more power than the standard association analyses, while on chromosome 18 only the CROSS test had more power. The significance of the effect on chromosome 6 was so strong that all methods had 100% power in this region.

**Table 1:** Type I error and power

	Type I error^a^			Power^b^		
		
Method	0.05	0.005	0.0005	Chr 6	Chr 11	Chr 18
Single-SNP association	0.051	0.0048	0.00057	1.0	0.7	0.1
Haplotype association	0.053	0.0052	0.00051	1.0	0.8	0.1
HSS	0.052	0.0046	0.00031	1.0	1.0	0.1
CROSS	0.051	0.0051	0.00061	1.0	1.0	0.4

^a^Type I results at three different significance levels were determined from the Replicates 1 to 10 of SNPs on all chromosomes except 6, 11, and 18.

^b^Power is determined as the fraction of replicates showing a significant result (p < 0.05/9187) in a 1-Mb region around SNP6_153 on chromosome 6, SNP11_389 on chromosome 11, and SNP18_269 on chromosome 18.

## Discussion

The results of our analyses on both the NARAC data and simulated genome-wide data show that the CROSS test is a powerful statistic for identifying disease loci. The CROSS combines information on differences in haplotype sharing, which are also used by the HSS, and on allele frequency differences, which are the basis of association analysis.

In the NARAC data, the CROSS test revealed a significant association (-log(*p*-value) = 6.6) approximately 4407 kb from the starting SNP. Odds ratios of 1.41 were observed at multiple SNPs in this region. A second suggestive peak was observed near 4863 kb (-log(*p*-value) = 4.1). This result was not significant after applying the conservative Bonferroni multiple testing correction, but because the significances outside of the associated region seemed to range up to a -log(*p*) of ~3, this result might indicate a relevant association as well. The HSS and the single-SNP association test peaked at the same locations as the CROSS, but these tests did not reach significance. Furthermore, the HSS and single-SNP association analyses were not specific enough because multiple signals at other positions reached the same significance level as the 4407 kb peak. The CROSS test did not identify a particular SNP as the causal one, which is inherent to the way the test was developed, but a candidate region as small as 20 kb could clearly be identified.

When applied to the simulated 10 k SNP array data, the HSS and CROSS tests showed significant results in the known candidate regions for rheumatoid arthritis, i.e., 6p21 and 11q23.1, and the CROSS was suggestive of association at 18q22.2 after Bonferroni correction. The type I errors of these haplotype-sharing methods were as expected and no significant associations in other regions were observed. Similar results were found by single-SNP and five-SNP haplotype association analysis, but the CROSS test showed more power in each associated region.

The strength of single-SNP association analysis was inferior to that of our two haplotype sharing methods, suggesting that the latter indeed contain more information. The HSS and CROSS showed more significant results and sharper peaks than the five-SNP haplotype analysis. This might imply that HSS and CROSS have a better mapping accuracy than a fixed-length or sliding-window haplotype method. This could result from the fact that HSS and CROSS use the variable length over which haplotypes are the same, whereas a sliding window method may use either too much or too little information. The finding that both the HSS and the CROSS test accurately identify the disease locus might seem to conflict with the results of the NARAC data, in which the exact locus could not be pinpointed. However, the SNP density of the NARAC data is about 80-fold higher than in the simulated data set and multiple highly correlated SNPs were expected to be associated causing broader peaks.

We did not investigate the robustness of the haplotype sharing methods to haplotype reconstruction errors or missing alleles. Although this analysis would be interesting and remains necessary, the focus of this paper was to introduce and describe the HSS and the CROSS test. The effects of phase ambiguities and missing alleles on the HSS and CROSS will be the topic of future work.

## Conclusion

The HSS and in particular the CROSS test show great promise for identifying and fine-mapping disease genes of complex diseases. They are useful for whole genome association screens because the analytical form of the HSS and the sequential randomization for estimating variance of the CROSS test renders them fast enough and hence practical to use even for marker densities of 500,000 SNPs/genome.

## Appendix

The mean haplotype sharing is a U-statistic: $U(k)=\frac{2}{N(N-1)}\cdot \sum_{i=1}^{N-1}\sum_{j=i+1}^{N}h(X_{i},X_{j};k)$. Denote the mean and variance of the sharing of two haplotypes at locus *k*, *h*(*X*_*i*_, *X*_*j*_; *k*), by *μ*_*k *_and *σ*_*k*_^2^, respectively. We state that, given a data set {*X*_1_,...,*X*_*N*_},

$$S{\left(k\right)}^{2}=S_{h}{\left(k\right)}^{2}-\frac{2}{N(N-1)}\cdot \sum_{i=1}^{N-1}\sum_{j=i+1}^{N}{\left(h(X_{i},X_{j};k)\right)}^{2}+U{\left(k\right)}^{2},$$

where

$$\begin{array}{c} S_{h}{\left(k\right)}^{2}=\frac{1}{\left(N-2)(N-3\right)}\cdot \{\left((N-2)(N-3)-2\right)\cdot \left(\frac{2}{N(N-1)}\sum_{i=1}^{N-1}\sum_{j=i+1}^{N}{\left(h(X_{i},X_{j};k)\right)}^{2}\right)\\ +4(N-1)\cdot \frac{1}{N}\sum_{i=1}^{N}{\left(\frac{1}{N-1}\sum_{\begin{array}{c} j=1\\ j\neq i \end{array}}^{N}h(X_{i},X_{j};k)\right)}^{2}-N(N-1)\cdot U{\left(k\right)}^{2}\}, \end{array}$$

is an unbiased estimate of the variance of a U-statistic. We will derive that *S*_*h*_(*k*)^2 ^is an unbiased estimate of var(*h*(*X*_*i*_, *X*_*j*_; *k*)) = *σ*_*k*_^2^, because from this finding, it simply follows that *S*(*k*)^2 ^is an unbiased estimator of var(U), as

$$\begin{array}{ll} E(S{\left(k\right)}^{2}) & =E(S_{h}{\left(k\right)}^{2})-\frac{2}{N(N-1)}\cdot \sum_{i=1}^{N-1}\sum_{j=i+1}^{N}E{\left(h(X_{i},X_{j};k)\right)}^{2}+E\left(U{\left(k\right)}^{2}\right)\\ & =\sigma_{k}^{2}-(\sigma_{k}^{2}+\mu_{k}^{2})+(\mathrm{var}(U(k))+\mu_{k}^{2})\\ & =\mathrm{var}(U(k)). \end{array}$$

It is simple to derive that $\frac{2}{N(N-1)}\cdot \sum_{i=1}^{N-1}\sum_{j=i+1}^{N}{\left(h(X_{i},X_{j};k)\right)}^{2}-\frac{4}{N(N-1)(N-2)(N-3)}\cdot \sum_{i=1}^{N}\sum_{j=i+1}^{N}\sum_{\begin{array}{c} l=1\\ l\neq i,j \end{array}}^{N}\sum_{\begin{array}{c} m=l+1\\ m\neq i,j \end{array}}^{N}h(X_{i},X_{j};k)\cdot h(X_{l},X_{m};k)$ is an unbiased estimator of *σ*_*k*_^2 ^(proof left to the reader). This estimator is of the order 4, as in the second term each haplotype pair is considered twice. To speed up the calculation, this estimator can be rewritten to an estimator of the order 2 as follows:

$$\begin{array}{l} \frac{2}{N(N-1)}\cdot \sum_{i=1}^{N-1}\sum_{j=i+1}^{N}{\left(h(X_{i},X_{j};k)\right)}^{2}-\frac{4}{N(N-1)(N-2)(N-3)}\cdot \sum_{i=1}^{N}\sum_{j=i+1}^{N}\sum_{\begin{array}{c} l=1\\ l\neq i,j \end{array}}^{N}\sum_{\begin{array}{c} m=l+1\\ m\neq i,j \end{array}}^{N}h(X_{i},X_{j};k)\cdot h(X_{l},X_{m};k)\\ =\frac{2}{N(N-1)}\cdot \sum_{i=1}^{N-1}\sum_{j=i+1}^{N}{\left(h(X_{i},X_{j};k)\right)}^{2}-\frac{4}{N(N-1)(N-2)(N-3)}\cdot [{\left(\sum_{i=1}^{N}\sum_{j=i+1}^{N}h(X_{i},X_{j};k)\right)}^{2}+\sum_{i=1}^{N}\sum_{j=i+1}^{N}{\left(h(X_{i},X_{j};k)\right)}^{2}\\ -\sum_{i=1}^{N}\sum_{j=i+1}^{N}\sum_{m=i+1}^{N}h(X_{i},X_{j};k)\cdot h(X_{i},X_{m};k)-\sum_{i=1}^{N}\sum_{j=i+1}^{N}\sum_{m=j+1}^{N}h(X_{i},X_{j};k)\cdot h(X_{j},X_{m};k)\\ -\sum_{i=1}^{N}\sum_{j=i+1}^{N}\sum_{l=1}^{i-1}h(X_{i},X_{j};k)\cdot h(X_{l},X_{i};k)-\sum_{i=1}^{N}\sum_{j=i+1}^{N}\sum_{l=1}^{j-1}h(X_{i},X_{j};k)\cdot h(X_{l},X_{j};k)]\\ =\left(1-\frac{2}{\left(N-2)(N-3\right)}\right)\cdot \frac{2}{N(N-1)}\cdot \sum_{i=1}^{N-1}\sum_{j=i+1}^{N}{\left(h(X_{i},X_{j};k)\right)}^{2}-\frac{N(N-1)}{\left(N-2)(N-3\right)}\cdot {\left(\frac{2}{N(N-1)}\sum_{i=1}^{N}\sum_{j=i+1}^{N}h(X_{i},X_{j};k)\right)}^{2}\\ +\frac{4}{N(N-1)(N-2)(N-3)}\cdot \sum_{i=1}^{N}\sum_{\begin{array}{c} j=1\\ j\neq 1 \end{array}}^{N}\sum_{\begin{array}{c} m=1\\ m\neq i \end{array}}^{N}h(X_{i},X_{j};k)\cdot h(X_{i},X_{m};k)\\ =\frac{1}{\left(N-2)(N-3\right)}\cdot \{\left((N-2)(N-3)-2\right)\cdot \frac{2}{N(N-1)}\cdot \sum_{i=1}^{N-1}\sum_{j=i+1}^{N}{\left(h(X_{i},X_{j};k)\right)}^{2}-N(N-1)\cdot U{\left(k\right)}^{2}\\ +4(N-1)\cdot \frac{1}{N}\sum_{i=1}^{N}{\left(\frac{1}{N-1}\sum_{\begin{array}{c} j=1\\ j\neq i \end{array}}^{N}h(X_{i},X_{j};k)\right)}^{2}\}\\ =S_{h}^{2}(k). \end{array}$$

## Competing interests

The author(s) declare that they have no competing interests.
